# Challenges of Antibiotic Formulations and Administration in the Treatment of Bloodstream Infections in Children Under Five Admitted to Kisantu Hospital, Democratic Republic of Congo

**DOI:** 10.4269/ajtmh.23-0322

**Published:** 2023-10-30

**Authors:** Bieke Tack, Daniel Vita, Emmanuel Ntangu, Japhet Ngina, Pathy Mukoko, Adèle Lutumba, Dina Vangeluwe, Jaan Toelen, Karel Allegaert, Octavie Lunguya, Raffaella Ravinetto, Jan Jacobs

**Affiliations:** ^1^ Department of Clinical Sciences, Institute of Tropical Medicine, Antwerp, Belgium;; ^2^ Department of Microbiology, Immunology and Transplantation, KU Leuven, Belgium;; ^3^ Department of Pediatrics, KU Leuven University Hospitals Leuven, Belgium;; ^4^ Saint Luc Hôpital Général de Référence Kisantu, Democratic Republic of Congo;; ^5^ Médecins Sans Vacances, Mechelen, Belgium;; ^6^ Department of Development and Regeneration, KU Leuven, Leuven, Belgium;; ^7^ Department of Pharmaceutical and Pharmacological Sciences, KU Leuven, Belgium;; ^8^ Department of Hospital Pharmacy, Erasmus University Medical Center Rotterdam, the Netherlands;; ^9^ Department of Microbiology, Institut National de Recherche Biomédicale, Kinshasa, Democratic Republic of Congo;; ^10^ Department of Medical Biology, University Teaching Hospital of Kinshasa, Democratic Republic of Congo;; ^11^ Department of Public Health, Institute of Tropical Medicine, Antwerp, Belgium;; ^12^ School of Public Health, University of the Western Cape, Cape Town, South Africa

## Abstract

Severe bacterial infections in children need prompt, appropriate antibiotic treatment. We report challenges observed within a prospective, cohort study on antibiotic efficacy in non-typhi *Salmonella* bloodstream infection (NCT04850677) in Kisantu district hospital (Democratic Republic of Congo). Children (aged > 28 days to < 5 years) admitted with suspected bloodstream infection (August 1, 2021 through July 31, 2022) were enrolled and followed until day 3 or discharge for non-typhi *Salmonella* patients. Antibiotics were administered to 98.4% (1,838/1,867) of children, accounting for 2,296 antibiotic regimens (95.7% intravenous, 4.3% oral). Only 78.3% and 61.8% of children were, respectively, prescribed and administered antibiotics on the admission day. At least one dose was not administered in 3.6% of children, mostly because of mismatch of the four times daily cefotaxime schedule with the twice-daily administration rounds. Inappropriate intravenous administration practices included multidose use, air-venting, and direct injection instead of perfusion. There was inaccurate aliquoting in 18.0% (32/178) of intravenous ciprofloxacin regimens, and thus administered doses were > 16% below the intended dose. Dosing accuracy of oral suspensions was impaired by lack of instructions for reconstitution, volume indicators, and/or dosing devices. Adult-dose tablets were split without/beyond scoring lines in 84.4% (27/32) of tablets. Poor availability and affordability of age-appropriate oral formulations contributed to low proportions of intravenous-to-oral switch (33.3% (79/237) of non-typhi *Salmonella* patients). Other quality issues included poor packaging, nonhomogeneous suspensions, and unsafe water for reconstitution. In conclusion, poor antibiotic products (no age-appropriate formulations, poor quality and access), processes (delayed prescription/administration, missed doses), and practices (inaccurate doses, [bio]safety risks) must be urgently addressed to improve pediatric antibiotic treatment.

## INTRODUCTION

Severe bacterial infections are a leading cause of death in children under age 5 years in low-resource settings, particularly in sub-Saharan Africa.^1^ A large portion of these deaths is attributable to bacterial bloodstream infections,^2^ especially if caused by antimicrobial resistant bacteria.^3^ Non-typhi *Salmonella* (NTS) is a predominant cause of bloodstream infections in children under age 5 in sub-Saharan Africa, with an average case fatality risk ∼17%.^4^^–^^7^ Since more than 2 decades, multidrug resistance (defined as coresistance to the previously used antibiotics ampicillin, co-trimoxazole, and chloramphenicol) is omnipresent in NTS bloodstream infections in sub-Saharan Africa.^5^ In addition, third-generation cephalosporin resistant and fluoroquinolone nonsusceptible NTS have emerged in the region and further limit antibiotic treatment options.^5^

Timely (within 1–3 hours after presentation) and correct administration of appropriate antibiotics to children with severe bacterial infections is essential for outcome and containment of antimicrobial resistance.^8^ Many studies on antibiotic use focus on appropriate antibiotic prescribing. However, access to and administration of antibiotics are also compromised in low-resource settings, particular in children under 5.^9^^,^^10^ Currently, insufficient access to quality-assured antibiotics still kills more children than antimicrobial resistance,^9^ and age-appropriate formulations are poorly available in low-resource settings.^11^^,^^12^ In children, age-appropriate formulations refer to pharmaceutical formulations that are adapted to the development of a child.^13^^,^^14^ Important aspects are dose flexibility without complex manipulation, swallowability, and palatability.^13^^,^^14^

As part of a prospective observational study (TreNTS study, registered on clinicaltrials.gov as NCT04850677) assessing the efficacy of different locally used antibiotic regimens to treat NTS bloodstream infection in hospital-admitted children in the Democratic Republic of Congo (DR Congo), we included a quality assessment of the available antibiotics. This assessment was done by a checklist for the visual inspection of physical appearance, package, and labeling of medicines, which, even if insufficient to detect all possible quality problems, can assist the health staff in detecting product quality problems.^15^ Using this checklist, we identified product-related shortcomings hampering adequate administration of antibiotics to children. Inspired by these findings, we identified hospital processes and practices that constituted further barriers to the antibiotic treatment.

The main objectives of the present work were to assess and explain the product-, process-, and practice-related factors that hampered appropriate antibiotic treatment in children in low-resource settings to illustrate how these factors are intertwined. As secondary objectives, we describe feedback of the on-site clinical and pharmacy staff and children’s caretakers on these factors, and compare costs of intravenous formulations, oral tablets, and oral suspensions. We further discuss recent improvements and potential ways forward. Evaluation of the choice for specific antibiotic regimens, ingredient quality, antibiotic coverage of targeted pathogens, and treatment outcomes are beyond the scope of this article.

## MATERIALS AND METHODS

### Study design, period, and setting.

Data are collected as part of a prospective observational study (TreNTS study, registered on clinicaltrials.gov as NCT04850677) assessing the efficacy of different locally used antibiotic regimens to treat NTS bloodstream infection in hospital-admitted children. Eligible children were aged > 28 days and < 5 years and admitted with suspected bloodstream infection to St. Luc Kisantu general referral hospital (Kisantu hospital). Criteria for suspicion of bloodstream infection were (history of) fever and severity signs (Supplemental Table 1). Children were enrolled 7 of 7 days, between 8 am and 4 pm on weekdays and between 8 and 12 am on the weekends. Children who arrived later were enrolled the next day.

Kisantu hospital is a district hospital located in a semirural health district (surface area 1,400 km^2^, population of 211.223 in 2022), 120 km south of Kinshasa in Kongo Central Province, DR Congo. It has 340 beds, of which 84 are pediatric beds. The pediatric ward has an average bed occupancy rate of 140%. On weekdays during office hours, the ward is staffed by three nonspecialist medical doctors, five nurses, two senior medical students, and > 10 junior medical students. On the weekend and after office hours, one medical doctor and two senior medical students cover the entire hospital and three (night) to five (weekend during day) nurses cover the pediatric service. When admitted, the child’s family pay a flat fee of 30,000 CDF (15 USD), which covers the consultation and admission fee, basic diagnostic tests, blood transfusion, and medication from the hospital pharmacy.^16^ After medicine prescription, the child’s caretaker is in charge to seek the prescribed medicines at the hospital pharmacy and keep the medicines at the child’s bedside until administration. Medicine administration mostly occurs during twice daily ward rounds (6 am and 6 pm) by the nursing staff. There are no standardized operating procedures about medicine administration on site. Procurement by the hospital pharmacy occurs partially via the local centralized medicines procurement center (Center d’Achat et Approvisionnement en Médicaments Essentiels de Kisantu) and partially via a private wholesaler. In case of nonavailability of medicines in the hospital pharmacy, caretakers are asked to buy medicines in private pharmacies outside of the hospital.

In DR Congo, 77% of the population lives below income poverty line (PPP $1.9/day), and resources for healthcare are very limited (health expenditure: 4.1% of GDP).^17^^,^^18^ Kisantu has a high, stable, and year-round *Plasmodium falciparum* malaria transmission that greatly increases during the rainy season (October through May).^19^ In Kisantu, high prevalence of malaria, anemia and malnutrition are associated with a high burden of NTS bloodstream infections, including frequent *P. falciparum* malaria–NTS coinfections.^20^^,^^21^ Blood culture service is embedded in routine patient care in Kisantu hospital since 2007, set up as surveillance by the Institut National de Recherche Biomédicale (Kinshasa, DR Congo) and Institute of Tropical Medicine (ITM; Antwerp, Belgium).^22^^–^^24^ Blood cultures are sampled free-of-charge on admission in children with suspected bloodstream infections. Incubation and work-up (identification and antibiotic susceptibility testing) are performed on-site according to previously published methods.^22^^–^^24^

### Data collection.

Data on patient characteristics, clinical evolution, blood culture results, and antibiotic prescription and administration were collected by a local, dedicated research team in electronic case-report forms (RedCap, Vanderbilt University, Nashville, TN). Each child’s clinical presentation, clinical follow-up, microbiology test results, and treatment were documented by research nurses and two medical doctors (J. N., E. N.) who were trained before study onset by the principal investigator. The research team did not participate in routine patient care, except for one research nurse (A. L.) who also worked in the pediatric intensive/emergency care ward and ensured that critically ill children were also enrolled.

Data on antibiotic administration were based on patients’ charts, observations from the research team and solicited feedback from the child’s caretaker. Due to anecdotally observed inappropriate practices in antibiotic administration in the first months of the study, the research team increased their attention to appropriate antibiotic administration over the study course. Therefore, recording of inappropriate administration techniques was initiated 4 months after the study start. If the research team observed major errors or inappropriate practices in antibiotic prescription or administration, they promptly discussed these with the attending clinicians and nurses. Other observed inappropriate practices, challenges, and potential solutions were discussed with the hospital staff in dedicated meetings during and after the study period.

Each last week of the month, a medical doctor of the research team (J. N.) together with the hospital pharmacist (P. M.) made an inventory of all antibiotics available in the hospital pharmacy that have been recommended in guidelines to treat NTS bloodstream infections.^5^ Hereafter, two products per month were selected from the inventory for quality visual checks. Quality checks were based on a previously published checklist for frontline healthcare workers to identify visually detectable quality problems.^15^ The selection was made by the principal investigator (B. T.) so that each antibiotic formulation from each manufacturer was checked at least once during the study period. After selection of a specific antibiotic product, the hospital pharmacist randomly sampled one package of the respective product. The checklist contained 26 simple yes/no questions regarding packaging, identification, traceability, and physical appearance.^15^

Furthermore, during the last 5 months of the study, a medical doctor of the research team (J. N.) and the hospital pharmacist (P. M.) also routinely checked during the inventory whether instructions for reconstitution, a dosing device, and a child-safe cap were present in (powder form) oral suspension formulations and whether a scoring line was present for all tablet formulations. Quality observations of the monthly visual check were supplemented by anecdotal observations of poor quality and quality use of antibiotics made by the research team during daily in-hospital follow-up of the enrolled children.

All data were verified and queried by an on-site dedicated data manager, the data manager from the ITM clinical trial unit and the principal investigator (B. T.). Study monitoring and supervision to ensure study quality and increase study insights were organized as weekly virtual meetings of the research team, site supervisor (D. V.), and principal investigator (B. T.) and regular site visits by the principal investigator (B. T.).

### Definitions and data analysis.

“Antibiotic regimen” refers in this article to a prescribed antibiotic, its dose, its duration, and its route of administration. “Initial antibiotic regimens” are defined as the first antibiotic regimens that were administered to the child after hospital admission. The local guidelines of Kisantu hospital recommend intravenous ceftriaxone (a third-generation cephalosporin) for empirical treatment of children with suspected bloodstream infection.^25^ Initiation of administration of more than one antibiotic on the same day was called “combination therapy.” If an additional antibiotic was added to the initial antibiotic regimen without stopping the initial regimen, this was called “association.” “Targeted antibiotic treatment” was defined as the antibiotic regimen administered after availability of blood culture results. For the present study, we considered the time of blood culture positivity and communication of the Gram-stain result as the time of availability of blood culture results. This choice was made because multidrug-resistant, third-generation cephalosporin-resistant *Salmonella* was responsible for the majority of bloodstream infections in children under age 5 in this setting (unpublished data). To limit morbidity and mortality, clinicians were recommended to switch to antibiotics that were presumed to be appropriate to treat these NTS (i.e., intravenous or oral ciprofloxacin or oral azithromycin) as soon as Gram-negative bacilli were isolated. It was presumed that NTS with pefloxacin resistance (detected by on-site disk diffusion testing) had intermediate ciprofloxacin susceptibility, as was observed during external reference testing (broth microdilution for determination of ciprofloxacin minimum inhibitory concentration [MIC]) from blood culture surveillance studies in Kisantu until 2017. Given the lack of access to meropenem, high-dose ciprofloxacin treatment was locally recommended for intravenous treatment of NTS with third-generation cephalosporin resistance and pefloxacin resistance. Unfortunately, a posteriori, external reference testing demonstrated full ciprofloxacin resistance (MIC values 1–2 mg/dL) in most pefloxacin resistant NTS. In retrospect, the use of ciprofloxacin to treat these NTS bloodstream infections was thus not appropriate. In this article, “tablets” refer to conventional, nondispersible tablets. “Oral suspensions” were available as powder or granules for reconstitution and rarely as readymade syrup.

Data analysis was performed in R studio with R version 4.2.2. Data were described by calculation of medians, interquartile ranges, and proportions. The first day of admission or the day of enrollment were counted as day 0 when duration of hospital admission or duration of study follow-up were calculated. When the duration of antibiotics was calculated, each day that at least one dose of antibiotics was administered was counted as 1 day. Pictures were assembled in PowerPoint (Microsoft Corporation, Redmond, WA) and direct identifiers of the stated medicine manufacturer were masked from the pictures.

### Ethics approval and consent to participate.

The study was conducted in accordance with the principles of the Declaration of Helsinki and international scientific standards. Written informed consent was given by the caretaker of each enrolled child. The study received ethical approval by the Ecole de Santé Public Kinshasa (134/2021), the Institutional Review Board of ITM (1483/21), and the Ethics Committee of Antwerp University (21/18/236). When poor-quality medicines were identified, by the pharmacovigilance committee of Kisantu hospital, the DR Congo pharmaceutical regulatory authority (ACOREP) was notified by e-mail.

## RESULTS

### Clinical characteristics and evolution of children with suspected bloodstream infection.

**Figure 1.**
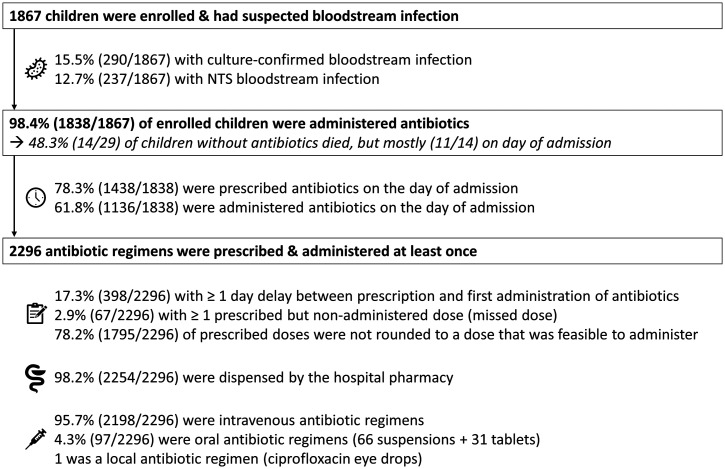
Overview of enrollment; blood culture positivity; and antibiotic timing, supply, and administration routes. NTS = non-typhi *Salmonella*.

During the 1-year study period (August 1, 2021 through July 31, 2022), 1,867 children with suspected bloodstream infection were enrolled. Enrollment occurred on the day of admission in 46.2% (862/1,867) and the day after admission in 53.8%. Children had a median age of 18 months (P25–P75: 10–31 months) and a median weight of 9.3 kg (P25–P75: 7.6–11.6 kg). Bloodstream infection was culture-confirmed in 15.5% (290/1,867) of enrolled children with suspected bloodstream infection (Figure 1). Non-typhi *Salmonella* were isolated from 81.7% (237/290) of children with culture-confirmed bloodstream infection. The remaining pathogens were *Klebsiella* spp. (*n =* 15), *Escherichia (para)coli* (*n =* 14), *Staphylococcus aureus* (*n =* 7), *Salmonella* typhi (*n =* 6), *Serratia* spp. (*n =* 2), *Acinetobacter* spp. (*n =* 3), *Citrobacter* spp. (*n =* 1), and *Candida* spp. (*n =* 1). Overall case fatality ratio was 6.9% (129/1,867). Case fatality rates of children with NTS bloodstream infection and culture-confirmed bloodstream infections with other pathogens were 24.9% (59/237) and 24.5% (13/70), respectively. Study follow-up occurred for < 3 days after enrollment in 6.2% (116/1,867) children, mostly due to early in-hospital death (80.2%; 93/116).

### Initial antibiotic treatment mainly relied on third-generation cephalosporins.

Antibiotics were administered to 98.4% (1,838/1,867) of children with suspected bloodstream infection. From the 29 children without antibiotic administration, 11 died on the day of admission. Among these 11 children, a blood culture was sampled in 10 children, and it confirmed a bloodstream infection in five of them. Three other children without administered antibiotics died: all three had a confirmed bloodstream infection, two of whom died on day 1 and one of whom died on day 2.

Almost all (99.5%, 1,828/1,838) children were administered their initial antibiotics intravenously. Initial antibiotic regimens were mostly (95.9%, 1,763/1,838) third-generation cephalosporins: ceftriaxone in 84.3% (1,549/1,838) and cefotaxime in 11.6% (214/1,838). Clinicians informally declared a preference for cefotaxime in case of meningitis or pneumonia due to (assumed) higher antibiotic concentrations at the infection site. Intravenous ciprofloxacin was administered to 2.8% (51/1,838) of children as the first-line antibiotic. Initial antibiotic treatment relied on combination therapy in 7.6% (139/1,838) of children, mostly with gentamicin or metronidazole (Table 1). Ten children had only oral antibiotic regimens as initial treatment (Table 1).

**Table 1:** Frequency table of initial antibiotic regimens

Antibiotic name	Ceftriaxone (C3G)	Cefotaxime (C3G)	Gentamicin	Ciprofloxacin	Metronidazole	Cloxacillin	Ampicillin	Azithromycin	Amoxicillin	Penicillin V	Erythromycin	Cefixime (C3G)
Ceftriaxone (C3G)	1,493		36		19		1					
Cefotaxime (C3G)		149	62†		3							
Gentamicin			1	2		1						
Ciprofloxacin				35*	15							
Metronidazole					3*							
Cloxacillin						8						
Ampicillin							2					
Azithromycin								3 PO				
Amoxicillin									2 PO			
Penicillin V										1 PO		
Erythromycin											1 PO	
Cefixime (C3G)												1 PO

C3G = ceftriaxone and cefotaxime are parenteral third generation cephalosporins, cefixime is an oral third generation cephalosporin; PO = oral administration. All antibiotics were administered intravenously, unless otherwise stated. Colors are graded according to frequency.

* All intravenous except for one child with oral administration.

† Combined with local ciprofloxacin eye care.

### Delayed antibiotic initiation and prescribed but nonadministered doses.

Only 78.3% of children were prescribed antibiotics on the day of their hospital admission (Figure 1). Furthermore, due to delays between prescription and administration (not further explored in this study), only 61.8% of children were effectively administered antibiotics on the day of admission (Figure 1). Antimalarials were prescribed slightly more often (84.8% of 1,220 children with positive malaria microscopy and antimalarial prescription) and administered 69.5% of 1,219 children with positive malaria microscopy and at least one antimalarial dose administered) on the day of admission. Overall, there was a delay of minimum 1 day between prescription and first administration for 17.3% (398/2,296) of antibiotic regimens.

In addition to delayed administration, 0.8% (*n =* 104) of 12.921 prescribed doses were reported as not administered, which corresponded to at least one missed dose in 2.9% (67/2,296) of antibiotic regimens and to at least one missed dose in 3.6% (66/1,838) of children. In 76.0% (79/104) of missed doses, caretakers reported that the nursing staff did not administer the antibiotic, which was mainly (91.1%, 72/79) cefotaxime. Cefotaxime has a three to quarter-daily dosing frequency, while routine administration rounds were organized only twice daily. In 20.2% (21/104) of missed doses, the antibiotic could not be administered because the caretaker(s) did not have the antibiotic bedside during the administration round, although the antibiotic was available in the hospital pharmacy. In 1.9% (2/104) of missed doses, there was no venous access. In 1% (1/104) of missed doses, the antibiotic was out of stock in the hospital pharmacy, and the caretakers could not afford to buy it on the private market.

Antibiotics of almost all (98.2%) regimens were dispensed by the hospital pharmacy (Figure 1). Regimens that relied for a minimum of 5% of prescriptions on out-of-hospital supply were intravenous cefotaxime (5.1%; 12/237), oral azithromycin (7.7%; 6/78), intravenous levofloxacin (80%; 4/5), oral co-trimoxazole (100%; 1/1), oral cefixime (20%; 1/5), and intravenous cloxacillin (9.1%; 1/11).

### Antibiotic regimens were rarely revised in children without confirmed bloodstream infection.

In 3.4% (62/1,838) of children, additional antibiotic regimens were associated to the initial antibiotic regimen. This was mostly (82%, 52/62) done 1 day after the start of the initial regimen. By the third day after antibiotic initiation, initial antibiotic regimens were changed to another regimen in 7.3% (134/1,838) of children. This was largely (79.1%, 106/134) confined to switching to targeted treatment in children with NTS bloodstream infection. Overall, 17.8% (332/1,867) of children were administered at least two antibiotic regimens during the 3-day study follow-up period. In two-thirds (66.2%; 956/1,838) of children, initial antibiotic regimens had a duration of more than 3 days. This implied that, upon prescription, caretakers picked up antibiotics for more than 3 days of treatment at the hospital pharmacy and that there was no incentive for reevaluation of children before antibiotics were continued beyond 3 days.

### Targeted treatment of NTS bloodstream infection relied on ciprofloxacin and azithromycin.

Most (89.4%, 212/237) children with NTS bloodstream infection were initially administered third-generation cephalosporins. From the children with NTS infection who were still alive and present in the hospital on the day of blood culture positivity (*n =* 204), 57.4% (117/204) were administered ciprofloxacin and 36.7% (75/204) were administered oral azithromycin as targeted treatment. Five children were administered intravenous levofloxacin after insufficient clinical improvement despite targeted ciprofloxacin treatment.

### Limited availability and affordability of oral suspensions as barriers for oral switch.

Most (94.8%, 1,734/1,838) children were exclusively administered intravenous antibiotics. Eight children were only treated with oral antibiotics, whereas 86 children were treated with oral and intravenous antibiotics, including one child who was also treated with local ciprofloxacin eye care. Children who received oral and intravenous antibiotics were mostly (91.9%, 79/86) children with NTS bloodstream infections who were switched from intravenous to oral targeted antibiotic treatment; that is, one-third (33.3%, 79/237) of children with NTS bloodstream infection had an oral switch.

Oral switch during targeted NTS therapy was largely (94.9%, 75/79) confined to azithromycin. Twenty children were treated with azithromycin targeted therapy formulated as tablets, and 56 children had it formulated as powder for suspension. Ciprofloxacin-targeted NTS therapy was oral in only 3 of 117 children. During the study period, there was only one liquid oral ciprofloxacin formulation registered in DR Congo (according to the latest publicly available list from the national pharmaceutical regulatory authorities for 2020^26^). Unfortunately, there was an 11-month nationwide stock rupture of this product, during which 500-mg tablets were the only oral ciprofloxacin formulation available. The three children with oral ciprofloxacin relied on these tablets, which had to be split in four. One child had co-trimoxazole as oral targeted therapy. This was formulated as powder for oral suspension and purchased from a pharmacy outside the hospital.

On the basis of the average wholesale price paid by the hospital pharmacy, tablets were up to 3 times cheaper per dose for a 10-kg child than (powder for) oral suspensions (Table 2). A 10-kg child could be treated for only 3 days with one vial of relatively expensive azithromycin suspension (Table 2), and caretakers reported the need for multiple vials as a barrier to complete the treatment when they had to buy it out-of-pocket outside the hospital. Nevertheless, when calculated per antibiotic day based on average medicine wholesale price only (excluding water for injection if not included in the package, catheter-related costs, etc.), oral antibiotic regimens were cheaper than intravenous regimens (Table 2).

**Table 2:** Comparison of packaging volumes and price per dose of intravenous vs. solid or liquid oral antibiotic formulations

Antibiotic	Formulation		Prescribed dose		Volume	Average wholesale price	
Active ingredient	Type	Concentration and package volume	Dosage regimen	Single dose (10-kg child)	Days/ package	Per package (USD)	Single day (10-kg child; USD)
Ceftriaxone	Powder + sterile water for IV injection	1,000 mg, single packaging	100 mg/kg per dose, q24h	1,000 mg	1	0.47	0.47
Cefotaxime	Powder to dilute for IV injection	1,000 mg, dispensed per vial	50 mg/kg per dose, q8h	500 mg	0.3	0.19	0.58
Cefixime	Oral tablet	200 mg, single packaging	10 mg/kg per dose, q12h	100 mg	1	0.05	0.05
	Powder for oral suspension†	250 mg/5 mL, 60 mL/vial			15	2.51	0.17
Ciprofloxacin	IV perfusion	200 mg/100 mL, 200 mL/vial	15 mg/kg per dose, q12h	150 mg	0.5	0.46	0.92
	Oral tablet	500 mg, single packaging			1	0.05	0.05
	Granulates for oral suspension*	250 mg/5 mL, 100 mL/vial			16.5	3.06	0.19
Azithromycin	Oral tablet	500 mg, single packaging	20 mg/kg per dose, q24h	200 mg	1	0.40	0.20
	Powder for oral suspension	100 mg/5 mL, 30 mL/vial			3	1.71	0.57
Ampicillin	Powder to dilute for IV injection	1,000 mg, dispensed per vial	50 mg/kg per dose, q8h	500 mg	0.3	0.19	0.58
Amoxicillin	Oral tablet	500 mg, single packaging	45 mg/kg per dose, q12h	450–500 mg	0.5	0.04	0.08
	Powder for oral suspension	250 mg/5 mL, 60 mL/vial			3	0.31	0.10
Co-trimoxazole	Oral tablet	400/80 mg, single packaging	20/4 mg/kg per dose, q12h	200/40 mg	1	No data	No data
	Ready-made oral suspension‡	200/40 mg/5 mL, 100 mL/vial			10	1.04	0.10

q8h–q24h = every 8 to 24 hours. Listed antibiotics are the antibiotics recommended for treatment of non-typhi *Salmonella* bloodstream infections that were available in the hospital pharmacy.^5^ Dosing regimens were according to the WHO AWaRe Handbook and cefixime according to the WHO Background document: “The diagnosis, treatment and prevention of typhoid fever” and to Nelson’s Pediatric Antimicrobial Therapy.^27^^–^^29^ Antibiotic days per package were calculated based on single-dose use of antibiotic vials for intravenous (IV) injection. Prices were average wholesale prices paid by the hospital at procurement and are only applicable for products dispensed by the hospital pharmacy.

* Ciprofloxacin granulates for oral suspension was only available during 1 of 12 months during the study period.

† Cefixime powder for oral suspension was only available during 5 of 12 months during the study period.

‡ Co-trimoxazole ready-made suspension was only available during 1 of 12 months during the study period.

### Prescribed antibiotic doses were not rounded to doses that were feasible to administer.

Antibiotic dosing and dosing frequency were relatively consistent among prescribers, except for gentamicin with a third of children being prescribed 5 mg/kg/day (neonatal dose) instead of 7.5 mg/kg/day (Supplemental Figure 1). For comparison of the dosing and dosing frequency of the antibiotic regimens to those proposed in the WHO AWaRe antibiotic book,^27^ we refer to Supplemental Figure 1. Intravenous ceftriaxone was dosed twice instead of once daily in half (49.8%, 747/1,554) of the children, of whom 74.0% (553/747) weighted more than 10 kg. This on-site practice was based on the content of one ceftriaxone vial (1,000 mg), that is, for a child weighing ≥ 10 kg a ceftriaxone dose of 100 mg/kg, two vials are needed, hence the local preference to divide in two doses per day.

Prescription was calculated according to weight but not rounded to doses that were feasible to administer in 78.2% (1,795/2,296) of antibiotic regimens—that is, they required a direct intravenous injection (previously called intravenous flush) accuracy of < 0.2 mL, an infusion or suspension volume accuracy of < 1 mL, splitting of nonscored tablets, or splitting of scored tablets in smaller pieces than supported by the scoring line. For example, a child of 3.8 kg was prescribed 28.5 mg of gentamicin (7.5 mg/kg/dose), which required the nurse to draw up 0.71 mL of gentamicin for subsequent dilution and administration, while only a syringe of 5 mL with 0.2-mL graduation was available for administration.

### Inaccurate dosing and at-risk manipulation of intravenous antibiotics.

**Figure 2.**
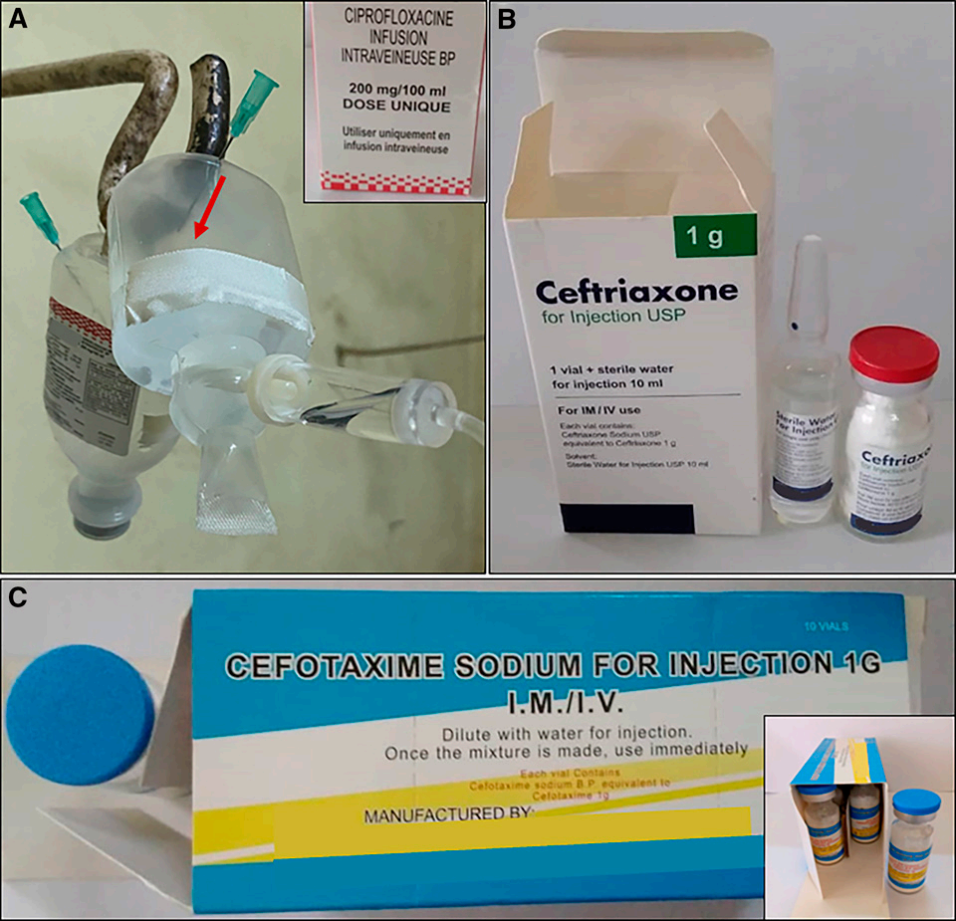
The challenges of intravenous antibiotic administration. (**A**) Multidose use of a single-dose (detail of package in upper right corner) ciprofloxacin perfusion vial (stated domestic manufactured product) with inaccurate aliquoting of the vial using tape as volume indicator (red arrow). Needles were inserted in the vial to overcome vacuum-induced poor perfusion flow but created a risk of vial contamination. (**B**) Package of ceftriaxone (imported product) containing a unit dose of sterile water for injection required for safe and correct reconstitution. (**C**) Packaging 10 cefotaxime vials (imported product) together without sterile water for reconstitution. To reconstitute the vials, nurses used 500 mL normal saline vials for multiple patients. Disclaimer: Direct identifiers of the antibiotic manufacturers were intentionally masked, but cases were separately reported to the national pharmaceutical regulatory authorities.

Data on the technique of intravenous antibiotic administration were recorded after December 2021. Intravenous ciprofloxacin administration (*n =* 148 regimens monitored after December 2021) faced several shortcomings. In half (50.7%; (75/148) of the cases, adult-dose ciprofloxacin perfusion vials designed for single use were inappropriately used as multidose vials by dividing them in two aliquots, one for the morning and one for the evening dose. In two other cases, ciprofloxacin vials were divided in three or four aliquots, respectively. Aliquoting was performed on a visual estimation with a tape as volume indicator (Figure 2), resulting in approximate dosing. Further, gross rounding of doses to half-full or full ciprofloxacin vials resulted in an administered dose that was a minimum of 5 mg/kg/day lower (≥ 16% lower) than the prescribed dose (20 or 30 mg/kg/day) in 21.6% (32/148) of intravenous ciprofloxacin administrations, and a minimum of 5 mg/kg/day higher (≥ 16% higher) in 4.1% (6/148) of cases (Supplemental Figure 2). Finally, in a quarter (37/148, 25.0%) of cases, in an attempt to administer the correct dose, nurses drew up the exact volume of ciprofloxacin from a perfusion vial with a syringe and (slowly) injected this directly intravenous (previously called intravenous flush) instead administering it correctly in a perfusion over 30 to 60 minutes.^30^

Similar practices were observed for intravenous metronidazole (aliquoting in 16% [5/31], direct intravenous injection instead of perfusion in 6.5% [2/31]), and intravenous levofloxacin (direct intravenous injection instead of perfusion in four of five cases).

Lastly, cefotaxime packages available in the hospital pharmacy rarely (no exact data available) contained a unit dose sterile water for injection (Figure 2). The same was sporadically observed for ceftriaxone. To administer these antibiotics, nurses used a 500-mL container of normal saline or glucose 5% water for a series of patients during an administration round. The date and time of first use was not noted on the fluid container.

### Inaccurate dosing and at-risk manipulation of oral antibiotics.

Two-thirds (68.0%, 66/97) of oral antibiotic regimens were prescribed as powder for suspension with the remaining third prescribed as tablets (Figure 1). Reconstitution of antibiotic powder into a suspension for oral administration was often inaccurate due to poor-quality packaging and labeling of the available products.

**Figure 3.**
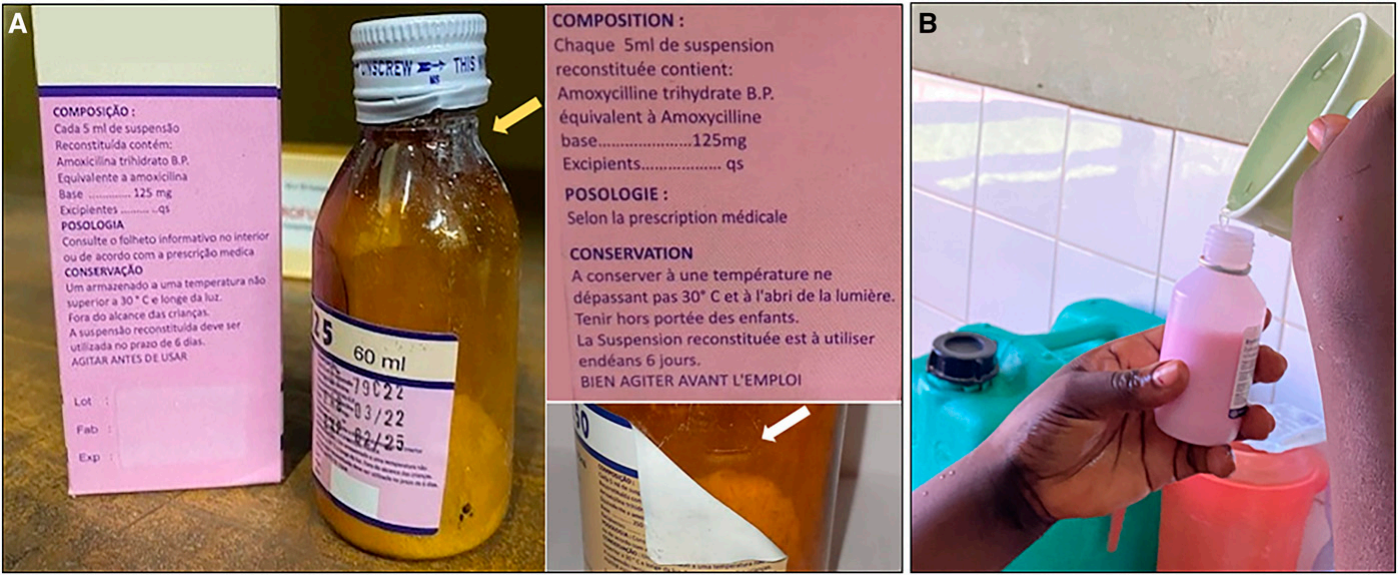
Inaccurate dosing and biosafety risks due to poor quality of oral antibiotic powder for suspension. (**A**) Poor packaging of amoxicillin powder for oral 125 mg/5 mL suspension (stated domestic manufactured product): poorly sealed with powder spill on outside of vial (yellow arrow), sticker label masking the volume indicator (detail in lower right corner with white arrow, picture from 250 mg/5 mL formulation), absent dosing device, absent instructions for reconstitution and no information on excipients (detail in upper right corner) and randomly printed lot number and manufacturing/expiry date. The information on the primary (on the bottle label) and secondary (on the carton box) package were the only known product characteristics because there was no separate leaflet with the summary of product characteristics. (**B**) In-hospital reconstitution of antibiotic powder for suspension by adding water stored in a 5-L jerry can until the volume indicator. Disclaimer: Direct identifiers of the antibiotic manufacturers were intentionally masked, but cases were separately reported to the national pharmaceutical regulatory authorities.

During the monthly visual quality check of antibiotics in the hospital pharmacy, we observed an almost universal absence of summaries of product characteristics (package leaflets were only included in the cefixime formulation for oral suspension and in an azithromycin formulation for oral suspension that was available for 1 month only). In the absence of leaflets with the summary of product characteristics, product information was limited to information on manufacturing, reconstitution, and storage that was printed on the primary (vial, tablet strip), and secondary (carton box) package, and excipients were not listed. Furthermore, we observed the absence of instructions for reconstitution of erythromycin and amoxicillin powder for oral suspension (Figure 3). The latter formulations were stated to be manufactured by the same domestic manufacturer. Furthermore, on the same products, the volume indicator, indicating the volume of water to add for reconstitution, was absent or masked by the label on the vial (Figure 3). In addition, we observed that samples from one batch of amoxicillin powder of 125 mg/5 mL and one batch of 250 mg/5 mL were poorly sealed with leakage of powder from the vial (Figure 3). Other packaging shortcomings were the absence of child-safe caps, and the use of stamps to mark the lot number, manufacturing, and expiry date on preprinted packages at a random site (Figure 3).

**Figure 4.**
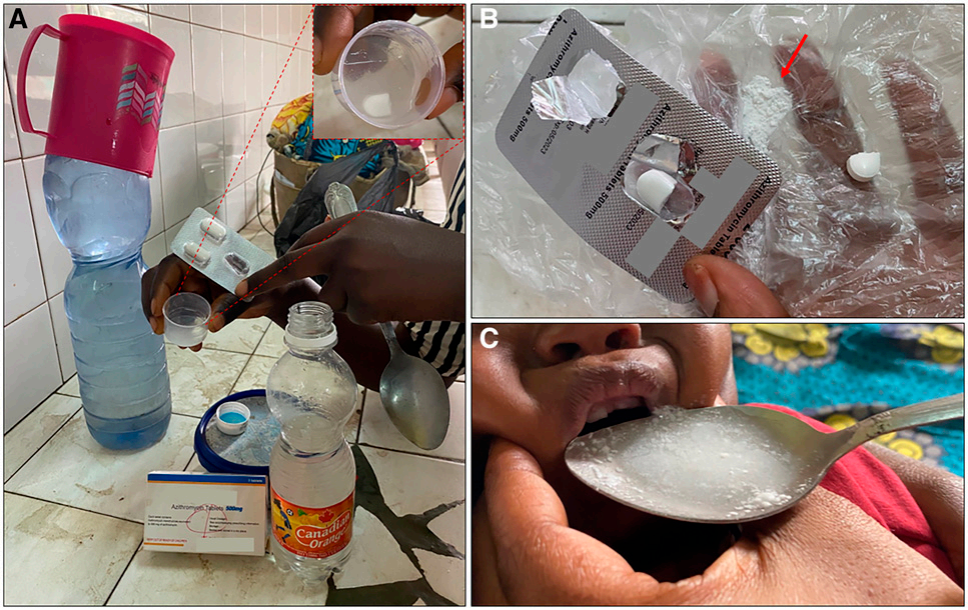
Administration of on-quarter of a 500-mg azithromycin tablet to a 12-month-old infant. (**A**) The mother halved the azithromycin tablet (imported product) on the scoring line and unsuccessfully tried to dissolve it in a volume of water that she kept in a recycled soft drink bottle. (**B**) In a next trial, the mother crushed the tablet bit per bit until she had the powder of approximately one-quarter of the tablet (red arrow), she kept the remnants of the tablet for a next dose. (**C**) The mother mixed the crushed tablet with the water (see panel A) and forced her infant to swallow it. Disclaimer: Direct identifiers of the antibiotic manufacturers were intentionally masked.

**Figure 5.**
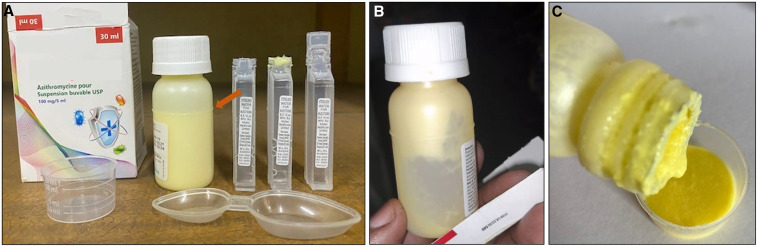
Poor suspension of azithromycin powder (imported product). (**A**) Packaging of azithromycin powder for oral suspension included water for reconstitution and dosing devices and was initially found acceptable by the visual inspection. After addition of two of three vials, the volume indicator was already reached (orange arrow); in the absence of a leaflet with the summary of product characteristics or detailed written reconstitution instructions on the package, this created confusion about the required water volume. We observed difficulties obtaining a homogenous suspension despite extensive shaking, and caking of the suspension after < 24 hours (**B**) with inability to reobtain a homogenous suspension (**C**). The excipients in this product were not listed. Disclaimer: Direct identifiers of the antibiotic manufacturers were intentionally masked, but cases were separately reported to the national pharmaceutical regulatory authorities.

Accurate dosing was further hampered by the absence of dosing devices in the package of 4/4 amoxicillin and 1/1 erythromycin powder for oral suspension formulations that were available in the hospital pharmacy. As a result, the vial cap (without volume indications) was often used to administer the suspension, resulting in inaccurate dosing but also biosafety risks. The most important biosafety risk of oral antibiotics, however, was the use of unsafe (not freshly boiled and cooled, no safe storage) water for reconstitution and administration (Figures 3 and 4). To prevent water-related biosafety risks, azithromycin powder for oral suspension was packaged with sterile water for reconstitution. However, the volume of water to add was unclear in the absence of specific reconstitution instructions (Figure 5). Furthermore, to obtain a homogenous suspension of this azithromycin formulation, more than 5 minutes of intense agitation was required and caking and homogeneity issues occurred (Figure 5).

Inaccurate dosing and biosafety risks were also observed for the use of tablet formulations intended for use in adults. They were used if oral suspension formulations were not available, not affordable, or rejected due to caking. To administer them to infants, all tablets were crushed and mixed with (potentially unsafe) water (Figure 4). Administration of the prescribed dose required splitting of adult-dose tablets (e.g., azithromycin or ciprofloxacin tablets of 500 mg) in less than half in 84.4% (27/32) of antibiotic tablets. Splitting was always performed manually (e.g., with the back of a spoon). There were no splitting devices. Leftovers from tablets after splitting were kept for next doses. Poor palatability and vomiting after administration were frequently observed for both tablets and oral suspensions.

## DISCUSSION

**Figure 6.**
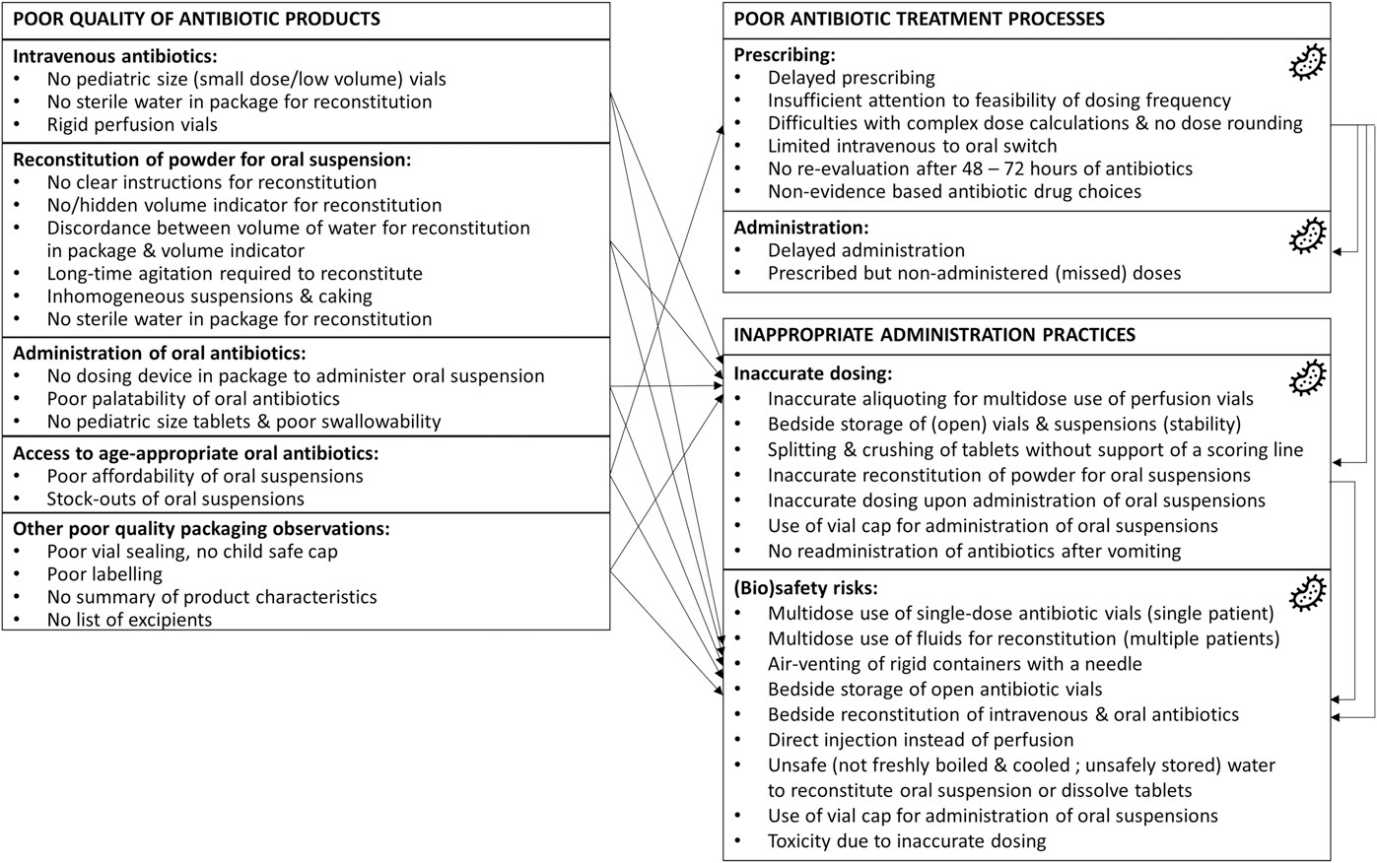
Summary of antibiotic treatment challenges and interrelations in children under 5 years in low-resource settings. The bacteria icon represents factors that drive antimicrobial resistance development and spread, arrows represent influencing factors.

In this study, we showed the multiple challenges of antibiotic treatment in children under age 5 years admitted with suspected of bloodstream infections in a low-resource setting in sub-Saharan Africa. Challenges occurred along the products (no age-appropriate formulations, quality, access, and affordability issues), processes (delayed prescription and administration, missed doses), and practices (inaccurate dosing, [bio]safety risks). They affected both intravenous and oral administrations and were interrelated (Figure 6).

### Inappropriate products, processes, and practices impact patient cure and safety and antimicrobial resistance.

The observed antibiotic treatment challenges impact patient cure and patient safety. Antibiotic treatment efficacy can be impaired by delayed initiation, inaccurate dosing, and missed doses. Multidose use of antibiotics and fluids for reconstitution is regularly done in low-resource settings, either because pediatric dose vials are unavailable or to save medication costs, but it poses a serious risk (and outbreaks) of healthcare-associated infections.^31^^,^^32^ Insertion of a needle to vent rigid antibiotic vials during administration and storing the open vial bedside further increase the risk of vial contamination.^33^ Furthermore, there is a risk of local venous site reactions if fluoroquinolones and metronidazole are directly injected instead of slowly perfused, as was observed in this setting, in an attempt to improve dosing accuracy in the absence of infusion pumps.^30^^,^^34^ Moreover, multidose use and bedside storage of fluoroquinolones and metronidazole perfusions can create stability issues (recommended storage out of direct light, below 25–30°C).^30^^,^^34^ Inaccurate dosing can cause toxicity, particularly for antibiotics with a narrow therapeutic window (e.g., aminoglycosides). The expected gains of the observed association of aminoglycosides to third-generation cephalosporins are small, whereas toxicity risks are substantial, particularly in the absence of therapeutic drug monitoring.^35^

Due to the high (bio)safety risks of intravenous antibiotics, an early switch from intravenous to oral antibiotics is recommended and can save lives in low-resource settings.^36^^–^^39^ Unfortunately, poor availability and affordability of oral suspensions hampered oral switch. In addition, biosafety of oral antibiotics was impaired because water for reconstitution and dosing devices (resulting in use of the vial cap for administration) were not included in the packaging.

At a population-level, inappropriate antibiotic products, processes, and practices fuel antimicrobial resistance. Development of antimicrobial resistance is favored by inaccurate dosing, and antimicrobial resistance spread is driven by healthcare-associated infections induced by the aforementioned biosafety risk practices. Moreover, some antibiotic regimens should be reserved to treat infections with multidrug resistant organisms; for example, levofloxacin is reserved for treatment of multidrug resistant tuberculosis.^40^

### Hospital pharmacist engagement is key to improve product quality and reconstitution.

Good collaboration between the clinical and pharmacy staff is essential to improve safety and dosing accuracy. Standardized tools such as the visual quality checklist used in this study can inform product selection upon procurement,^15^ for example, to select oral suspensions with clear instructions for reconstitution and with a dosing device included. Moreover, procurement of pediatric dose vials (low dose/small volume) and packaging including single-use sterile water for reconstitution can prevent multidose use, and flexible instead of rigid containers can bypass the need for air-venting.^33^^,^^41^^,^^42^ Correct reconstitution of oral suspensions can be most appropriately organized by the hospital pharmacy—that is, performed in a room that is separated from patient care with a correctly measured volume of safe (e.g., freshly boiled) water stored in a safe container.^43^^–^^46^ Reconstitution at the hospital pharmacy also allows direct detection of reconstitution problems and thus facilitate pharmacovigilance, optimization of product selection for procurement, and reporting of problems to the supplier and to the national regulatory authority. The pharmacy staff should also provide guidance on correct storage and on splitting and crushing of tablets, when oral suspensions are not available or affordable.^46^^,^^47^

To improve pharmacovigilance and to boost detection and reporting of substandard formulations, the hospital pharmacist could consider applying the visual checklist also to the products that must be purchased by the caretakers outside the hospital, for hospitalized children. This would seem a useful approach, considering that the private and informal sector in DR Congo are surely not exempted from the risk of substandard or falsified products. However, it is unlikely that there would be sufficient time for this additional activity in a routine setting.

### Ward-based interventions to improve prescription, reconstitution, and administration.

Pediatric wards in low-resource settings are often overcrowded and understaffed, and a high nursing workload has been associated with medication errors and healthcare-associated infections.^35^^,^^48^^–^^51^ Furthermore, staff misconceptions may hamper rational antibiotic use, as was illustrated here by the prescribers’ preference for cefotaxime over ceftriaxone, which lead to the hidden consequence of missed doses. Finally, also caretaker’s misconceptions have an impact, for example, oral switch is hampered by a high demand for “strong,” or intravenous medication.^41^^,^^52^^–^^54^

Internal reorganization of triage and initial evaluation can accelerate antibiotic prescribing and make the process from prescription to (first) administration more accurate and efficient. Administration should follow immediately and not be postponed until the next administration round.^55^ To reduce the time to administration, it might also be necessary to set up a small stock of frequently used antibiotics in the emergency ward (e.g., ceftriaxone) to overcome antibiotic supply and dispensing-related delays. Furthermore, prescribing antibiotics upon presentation for maximum 3 days can be considered as intervention to facilitate reevaluation and antibiotic revision after 48–72 hours, although caution for unwanted treatment interruptions is warranted.^56^

Next, standard operating procedures, staff training, and quality monitoring are required to improve antibiotic prescribing, reconstitution, administration, and storage. Standard operating procedures should be tailored to the setting (e.g., when multidose vials cannot be avoided), use of one antibiotic vial per patient that is stored away from the patient environment without inserted needle can be recommended.^57^ For off-label multidose use of reconstitution fluids, the date and time of first use should be written on the product, products should be stored as recommended by the manufacturer and used for maximum 24 hours, normal saline must be preferred over glucose-containing fluids, and access points should be disinfected.^58^ Training should target common misconceptions of prescribers but also focus on caretaker’s education.^51^ Furthermore, prescribers must consider minimal dosing frequencies, dose rounding, feasibility of antibiotic perfusion, and early switch to oral antibiotics to minimize errors and inappropriate administration practices in busy wards.^59^^–^^61^ Finally, compliance to good practices must be enhanced by nursing leadership, regular refresher trainings, and monitoring.^41^^,^^51^

### Research and development: adapt antibiotic formulations to the needs of children in low-resource settings.

Many of the observed challenges in antibiotic administration could be prevented by adapting antibiotic formulations to the needs of children, including babies and infants, in low-resource settings. Less than a third of the oral medicines on the WHO Model List of Essential Medicines for Children are appropriate for children under age 5.^13^ The WHO-hosted, multistakeholder network Global Accelerator for Pediatric Formulations has added antibiotics to their target medicines in 2022.^62^ In March 2023, they have marked their first steps with the publication of a priority list of antibiotics for pediatric medicines optimization, including oral azithromycin, amoxicillin-clavulanic acid and nitrofurantoin, and parenteral cefiderocol.^63^

Oral formulations must be swallowable and flexible to dose to avoid splitting and crushing of tablets.^13^^,^^14^^,^^43^^,^^46^^,^^47^ Pediatric antibiotics are therefore mostly formulated as (powder for) oral suspensions.^64^ However, inaccurate dosing and biosafety risks are well-known disadvantages of oral suspensions, as are palatability, solubility, chemical instability requiring refrigeration, antimicrobial contamination risk, high dose volumes, the need for dosing devices, and bulky package volumes.^43^^,^^65^^–^^67^ Therefore, quality-assured oral antimalarials are now widely available as dispersible, sweetened tablets with pictorial instructions printed on the package.^68^^–^^70^ Dispersible tablets are also available for amoxicillin, but uptake is limited despite improved adherence.^64^^,^^71^ Recently, some quality-assured antiretrovirals have been formulated as multiparticulates or minitablets.^72^ These very small (diameter < 0.05 mm and 1–3 mm, respectively) solid formulations allow flexible dosing and are relatively easy to swallow.^72^ Like conventional tablets, their stability is good, their packaging volume is low, and they can be coated to mask taste or modified release.^72^ Dispensing difficulties are currently a major limitation but dispensing devices are being developed.^72^ Also for existing formulations, packaging should be optimized: low dose/small volume pediatric vials, flexible bags instead of rigid perfusion vials, clear instructions for reconstitution, and inclusion of sterile and volume-adapted reconstitution fluid and dosing devices in the package.^33^^,^^42^^,^^43^

Finally, nonpharmaceutical innovations are important to improve dosing accuracy. Complex dose calculations can be simplified by mobile applications or by weight-banded dosing as currently proposed for oral antibiotics in the WHO AWaRe antibiotic book.^27^ Access to syringe and infusion pumps is limited in low-resource settings despite their inclusion in the WHO list of priority medical devices for pediatric healthcare.^73^^,^^74^ To improve access, pumps must be affordable. As for other medical products, lower production costs and thus lower prices could probably be achieved by increasing production. In other words, a higher demand could trigger economies of scale and increase affordability.^75^^,^^76^ The pumps should also be adapted to low-resource settings (e.g., frequent power cuts and low technical support).^74^ A target product profile for syringe pumps for neonatal care has been developed.^77^

### Regulation and stakeholders’ commitment.

To advance universal health coverage for children, the stakeholders in health and pharmaceutical systems, including but not limited to national regulatory authorities, national procurement centers, health insurers, and external donors, must develop strategies to improve access to affordable, quality-assured, age-appropriate antibiotics.^78^ To the best of our knowledge, poor-quality antibiotics have been most frequently reported in Africa.^79^ The most frequently reported quality problems relate to inadequate content in the active product ingredient and inadequate dissolution profile, including for the reports available for the DRC.^79^^–^^84^ However, packaging-related quality failures were also observed.^79^ Unfortunately, prospective medicines quality surveys rarely focus on pediatric medications; therefore, problems with (the lack of) pediatric formulations may be underreported in the scientific literature. Between 2022 and 2023, three major incidents due to the contamination of oral syrups with ethylene- and diethylene-glycol (excipients) caused the death of at least 300 children across The Gambia, Indonesia, and Uzbekistan, tragically highlighting the lack of protection for children in resource-limited settings from substandard medicines.^85^^–^^88^

In our study, which relied on a visual assessment for the detection of quality problems, most poor-quality packaging and labeling observations seem to reflect negligent or cost-saving manufacturing practices (e.g., poor and non–child-safe sealing or the absence of volume indicators, dosing devices, and sterile water for reconstitution). Lower manufacturing costs increase the profit for the manufacturer, and they may translate into lower average wholesale prices, which is apparently advantageous for the healthcare system. However, substandard products will finally result in higher healthcare costs due to limited efficacy, safety, and the risk of antimicrobial resistance.^89^ This cost balance should inform procurement decisions at the hospital, centralized procurement services, and national levels.^78^ Furthermore, poor practices in the packaging and labeling of medicines could also be an indicator of poor practices in other key steps of the pharmaceutical cycle, including manufacturing. These could cause other major quality problems, such as underdosing and poor bioavailability, which cannot be detected visually but will have a negative impact on health outcomes.

National regulatory authorities are responsible to ensure the efficacy, safety, and quality of all medicines manufactured or imported in a country, including antibiotics. However, with some notable exceptions, most national regulatory authorities in low-resource settings are still under-resourced, and their level of “maturity,” as assessed on a 1 to 4 scale with the WHO Benchmarking Tool, is still 1 or 2, which is weak.^78^^,^^89^ To strengthen their capacity to assess manufacturers and products, to grant licenses and market authorizations, and to carry out other essential regulatory functions, immature regulatory authorities need to be supported by a strong political commitment. Regional initiatives for the harmonization of pharmaceutical regulation have also proven to motivate regulatory strengthening, and given that DR Congo joined the East African Community (EAC) in 2022, it is hoped that ACOREP could benefit from the advancement of the EAC Regulatory Harmonization.^90^ In the short term, immature regulators could rely on granting marketing authorizations, the opinion of stringent regulatory authorities, or the WHO prequalification of medicines.^89^^,^^91^ Unfortunately, there is no WHO prequalification for antibiotics, even if a survey among NGOs indicated antibiotics as a priority field for its expansion.^89^^,^^91^

In our study, we observed quality concerns both in antibiotics stated as imported and as domestically manufactured. This highlights the importance of more stringent regulatory oversight along all the key steps of manufacturing, importing and distributing medicines in a low-resource setting.^92^ The quality issues reported here were detected with simple visual checks, or via feedback from caretakers. This experience illustrates that even if limited to visually detectable problems, postmarketing surveillance can be built at hospital level, with multiple advantages: increased awareness of pharmacists and nurses, improved pharmacovigilance and reporting, and reorientation of local procurement practices.^15^^,^^78^ However, risks can be truly minimized only in the presence of a strong political commitment to reinforce national regulatory capacities and to enforce regulatory and legal sanctions for those who manufacture or supply substandard medicines, including in case of substandard labeling and packaging.^93^ For example, the U.S. Food and Drug Administration issued multiple batch recalls due to inaccurate dosing information and devices.^94^

### Strengths, limitations, and generalizability.

Previous studies on appropriate antibiotic treatment in low-resource settings focused on poor-quality products, antibiotic prescribing, accurate dosing, or biosafety.^32^^,^^41^^,^^42^^,^^44^^–^^47^^,^^79^^,^^95^ Here, conversely, we provide a bottom-up and comprehensive overview of product, process, and practice challenges faced during antibiotic treatment of bloodstream infections in children under 5 in a low-resource setting. The combination of data collection by a dedicated, trained, and supervised research team, with regular transparent and open-minded discussions with hospital staff and children’s caretakers, facilitated insights in the relations between the observed challenges.

However, the observation-driven and interactive nature of the reported findings biased their frequency (e.g., the observation of inaccurate ciprofloxacin aliquoting triggered the researchers’ attention for this practice), but it also increased the practice of direct injection instead of perfusion. The frequency of the reported challenges is probably underestimated because it is based on patient files, parental report, or bedside leftovers of antibiotics, but not on direct observation. Other studies conducted in sub-Saharan Africa have reported missed antibiotic doses in one in three patients.^61^^,^^96^ Furthermore, differences between prescribed and administered doses reported in Supplemental Figure 2 were not measured but estimated based on the observation of aliquoting.

Finally, Kisantu hospital may represent a best-case scenario due to its relatively solid financial system, international support, and location relatively close to Kinshasa; this is reflected, for example, in the fact that almost all antibiotics were available at and could dispensed by the hospital pharmacy. The clinical study and surveillance setting probably also improved patient care. Generalizability is also limited by the focus on NTS bloodstream infections, with follow-up of children without NTS limited to 3 days. It is likely that antibiotic choice, dosing, switch to oral antibiotics, and duration were more variable in children without NTS (e.g., due to a lack of guidance to target or stop antibiotics in a setting with few diagnostic facilities). Furthermore, challenges that are specific to antibiotics that were not used to treat NTS bloodstream infections will be underreported. Nevertheless, due to their high burden in children under 5 years, severity, and antimicrobial resistance profile, NTS bloodstream infections are a good illustration of barriers to appropriate antibiotic treatment of severe bacterial infections in children.

Although we did not perform a root-cause analysis of the observed challenges, two sequential sessions were organized at the hospital-level to present and discuss the results and to identify action points. The first session was in restricted group with presence of the principal investigator (B. T.), site supervisor and medical director of Kisantu hospital (D. V.), the medical doctors of the study team (J. N., E. N.), two medical doctors from the pediatric ward, three nursing staff (including hospital head and pediatric head of nursing), and the hospital pharmacist. During the second session, the action points from the first session were discussed with the nursing staff and medical doctors from the pediatric ward. Action points were focused on acceleration of antibiotic initiation (need for attitude change and proactive communication, no need to wait for laboratory results, starting an antibiotic is as urgent as starting antimalarials, risk–benefit of an emergency stock), multidose vials (stop multidose use of perfusion vials, dispense all intravenous antibiotics with unit dose of sterile water for reconstitution, explore market availability of infusion pumps), quality and reconstitution of oral suspensions (stop procurement of substandard products, reconstitution on pharmacy level), and resolution of misconceptions hampering rational prescribing (oral switch, prescription of initial antibiotics for maximum 3 days, association and dose of gentamicin, frequency of ceftriaxone, levofloxacin use). These sessions were highly appreciated by the hospital staff.

## CONCLUSION

Antibiotic treatment in hospital-admitted children under age 5 years faced multiple challenges along product (no age-appropriate formulations, quality, and access issues), processes (delayed prescription and administration, missed doses), and inappropriate practices (inaccurate doses, [bio]safety risks). These challenges originated from the constraints of a low-resource, pediatric setting and were interrelated. Short- and long-term corrective measures need to be articulated at local, national, and supranational level to improve antibiotic product quality and appropriate use at hospital pharmacy- and ward-level, by engaging all relevant stakeholders including those in regulation, health system financing, and research and development.

## Financial Disclosure

This research was funded by the Belgian Directorate of Development Cooperation and Humanitarian Aid (DGD) through Framework Agreement between the Belgian DGD and the Institute of Tropical Medicine, Belgium. B.T. has a scholarship from Research Foundation Flanders (FWO, 1153220N and 1153222N) and received a research grant for the study “Treating Non-typhoidal *Salmonella* Bloodstream Infections in Children Under-five in DR Congo: a Cohort Study (TreNTS)” from the European Society of Clinical Microbiology and Infectious Diseases (ESCMID) in 2020. Part of the blood culture surveillance activities were supported by the Bill and Melinda Gates Foundation project OPP1127988 funded to International Vaccine Institute. This study has also received funding from the European Union’s Horizon 2020 research and innovation program under the Vacc-iNTS project, grant agreement No. 815439. The funders had no role in study design, data collection and analysis, decision to publish, or preparation of the manuscript.

## Supplemental files

10.4269/ajtmh.23-0322 Supplemental Materials
