# Supplementary material for: Challenges of Antibiotic Formulations and Administration in the Treatment of Bloodstream Infections in Children Under Five Admitted to Kisantu Hospital, Democratic Republic of Congo
Source: Am J Trop Med Hyg. 2023 Oct 30;109(6):1245–59. doi: 10.4269/ajtmh.23-0322 (PMC10793067; doi:10.4269/ajtmh.23-0322)

Supplementary Materials

Supplementary Table S1. Criteria defining suspected bloodstream infection. If children fulfilled both criteria when they arrived at the hospital, bloodstream infection was suspected and a blood culture was sampled and worked up

**CRITERIUM 1: Fever or Hypothermia**			
	Rectal	Axillary	Tympanic
Fever upon presentation	> 38.0 °C	> 37.5°C	> 37.5°C
Hypothermia upon presentation	< 36.0 °C	≤ 35.5°C	≤ 35.5°C
Subjective fever in the past 48 hours (as reported by the caretaker)			
**CRITERIUM 2: Minimum 1 Severity Sign Present**			
Quick SOFA score ≥ 1	Hypotension		
	Confusion		
	Increased respiratory rate		
Suspicion of severe localized infection	Pneumonia		
	Meningitis		
	Osteomyelitis		
	Complicated urinary tract infection		
	Abscess		
	Severe skin or soft tissue infection		
	Abdominal infection		
Suspicion of other severe systemic infection	Severe malaria		
	Typhoid fever		

Supplementary Figure S1. Prescribed daily dose and dose frequency of antibiotics that were prescribed to minimum five children. Dosage regimens recommended in the WHO AWaRe antibiotic book were added for comparison. Each dot in the plot represents one prescription. Abbreviations: q6h – q24h = every 6 – 24 hours.

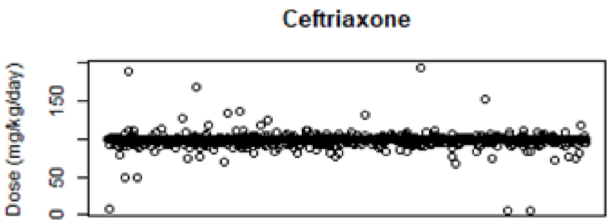

Dosing frequency	Dosing in AWaRe antibiotic book
Q24h: 807/1554 (52%)<br>Q12h: 747/1554 (48%)	80 -100 mg/kg/dose, q24h<br>= 80 – 100 mg/kg/day

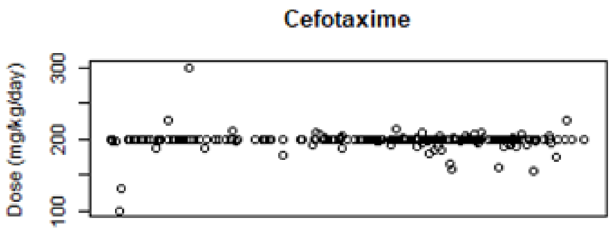

Dosing frequency	Dosing in AWaRe antibiotic book
Q6h: 237/237 (100%)	50 mg/kg/dose, q8h<br>= 150 mg/kg/day

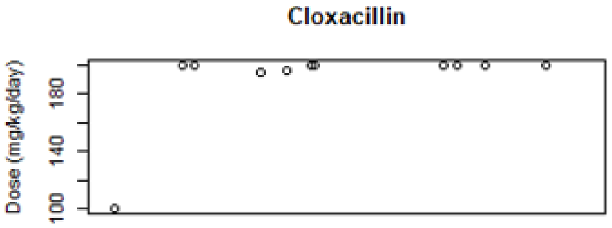

Dosing frequency	Dosing in AWaRe antibiotic book
Q8h: 1/11 (9%)<br>Q6h: 10/11 (91%)	25 mg/kg/day, q6h<br>= 100 mg/kg/day

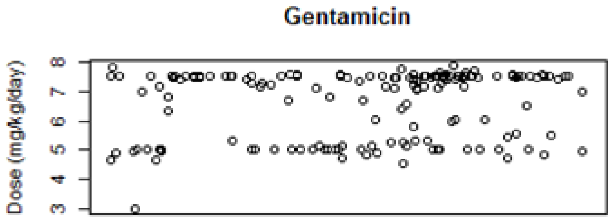

Dosing frequency	Dosing in AWaRe antibiotic book
Q24h:160/161 (99%)<br>Q12h: 1/161 (0.6%)	7.5 mg/kg/dose, q24h<br>= 7,5 mg/kg/day

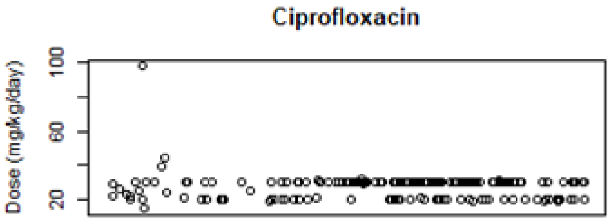

Dosing frequency	Dosing in AWaRe antibiotic book
Q12h: 180/180 (100%)	15 mg/kg/dose, q12h<br>= 30 mg/kg/day

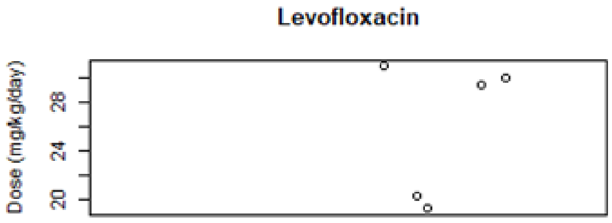

Dosing frequency	Dosing in AWaRe antibiotic book
Q12h: 5/5 (100%)	Not recommended

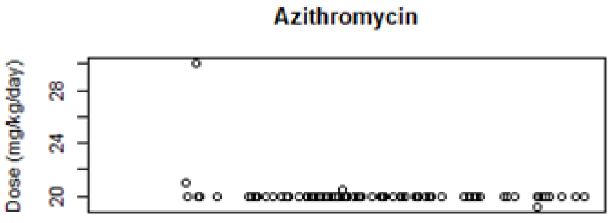

Dosing frequency	Dosing in AWaRe antibiotic book
Q24h: 78/78 (100%)	20 mg/kg/dose, q24h<br>= 20 mg/kg/day

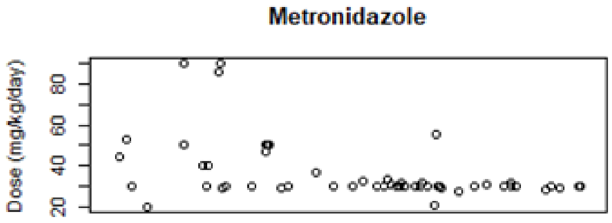

Dosing frequency	Dosing in AWaRe antibiotic book
Q12h: 1/54 (2%)<br>Q8h: 53/54 (98%)	7.5 mg/kg/day, q8h<br>= 22.5 mg/kg/day

Supplementary Figure S2. Boxplot displaying the differences between prescribed and administered doses of ciprofloxacin according to dosage regimen (20 mg/kg/day: *n* = 45, 30 mg/kg/day: *n* = 103) and administration technique**.**

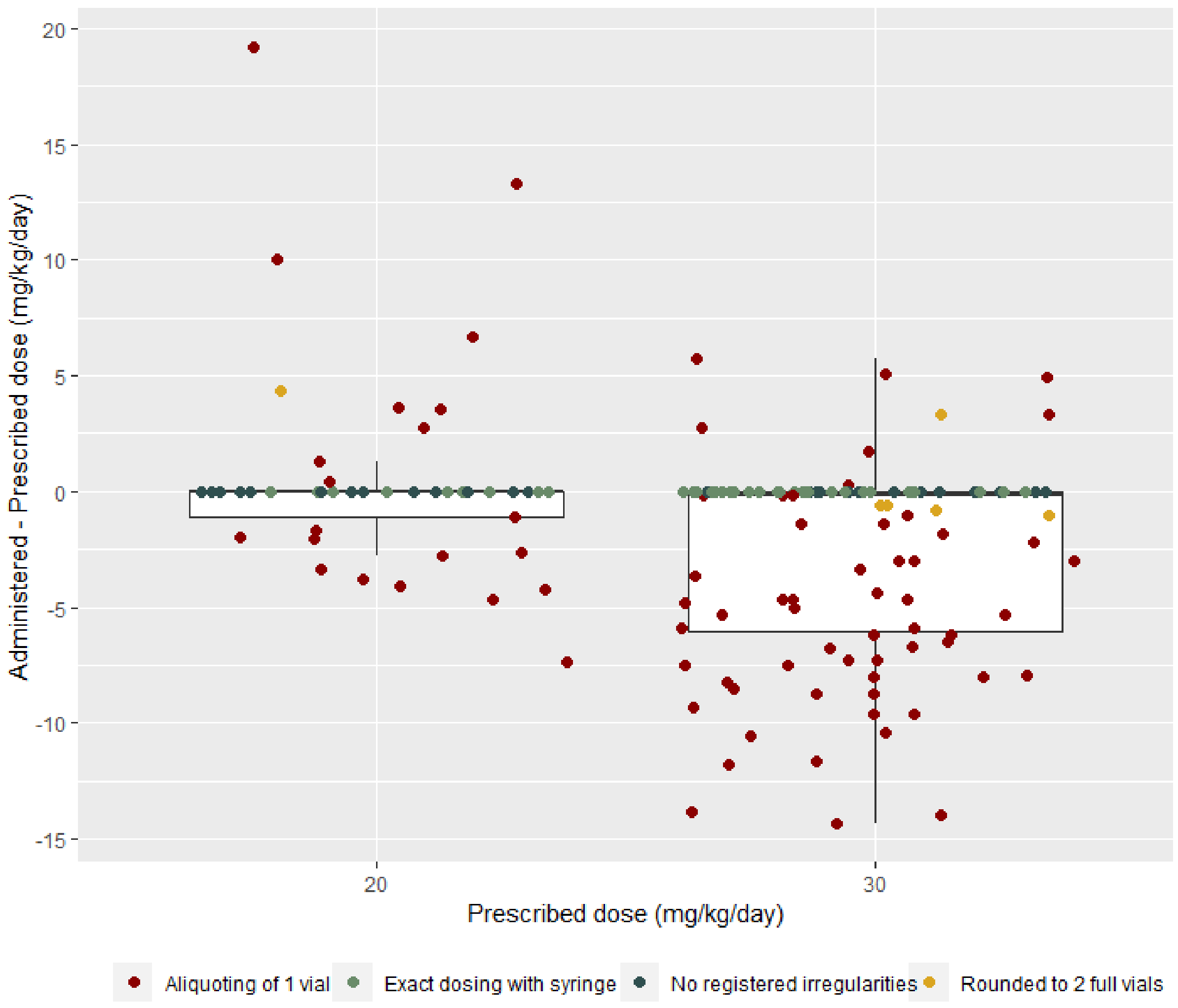

Supplement: Supplemental Materials [file tpmd230322.SD1.pdf]
